# Advancing translational science through biostatistics, epidemiology, and research design consultations: A multi-perspective evaluation of the Georgia CTSA BERD program

**DOI:** 10.1017/cts.2026.10748

**Published:** 2026-05-06

**Authors:** Gaurav Rajgopal, Nicole Llewellyn, John Hanfelt, Julia Wrobel, Eric Nehl

**Affiliations:** 1 Georgia Clinical & Translational Science Alliance, Emory University School of Medicine, Atlanta, GA, USA; 2 Emory University Rollins School of Public Health, Atlanta, GA, USA

**Keywords:** Biostatistics, multi-perspective evaluation, consultation effectiveness, clinical and translational science award, BERD program

## Abstract

A multi-perspective evaluation of a Biostatistics, Epidemiology, and Research Design (BERD) program was conducted within an NIH Clinical and Translational Science Awards hub. The program provides individual and group consultations, an educational forum event, and pilot and conference grants. Surveys from consultants and consultees following 62 BERD consultations spanning one year across four institutions assessed satisfaction, effectiveness, and expectations. Respondents expressed strong satisfaction, intent to recommend consultations, and many noted that the support strengthened their study design and grant preparation. Consultants highlighted the need for additional BERD resources to strengthen pre-consultations guidance, sustained follow-up, and mentoring for early-career investigators.

## Introduction

Biostatistics, Epidemiology, and Research Design (BERD) programs, a foundational component of the National Institutes of Health (NIH) funded Clinical and Translational Science Awards (CTSA) program, provide essential support for clinical and translational science (CTS). The Georgia Clinical and Translational Science Alliance (Georgia CTSA) is a multi-institutional NIH-funded partnership among Emory University (Emory), Morehouse School of Medicine (MSM), Georgia Institute of Technology (Georgia Tech), and University of Georgia (UGA), established to accelerate CTS through resources, education, and community engagement across the state of Georgia and beyond. BERD program members – primarily biostatistics and epidemiology faculty at the four Georgia CTSA institutions – are funded by CTSA hubs to provide research support that advances CTS. Their distribution across partner institutions enables investigators to access complementary expertise and tailored support, leveraging the strengths of the broader CTSA landscape. The Georgia CTSA BERD program fosters cross-disciplinary collaboration and serves as a core pillar of the CTSA hub. BERD consultants consult on study design, analytical planning, and statistical analysis to promote research rigor, transparency, and reproducibility [1]. BERD units within CTSA institutions across the nation vary significantly in size, staffing, and workload, typically comprising of doctoral level biostatisticians and epidemiologists who manage substantial volumes of investigator-initiated CTS consultations annually, underscoring their critical role within the translational science infrastructure [2]. More broadly, biostatistics plays an important role in advancing CTS, by improving research quality, specifically in grant development and early-stage project design, and is instrumental in supporting investigators in navigating the research process [3,4].

The Georgia CTSA BERD program offers services across Emory, MSM, Georgia Tech, and UGA to enhance research rigor and support high-quality translational research. These services include an annual BERD Research Forum, as well as participation in CTSA-wide programs such as Studio Consultations and conference grant mechanisms. Through these activities, BERD provides methodological expertise that supports investigators at multiple stages of research. The BERD program also contributes to research development by awarding methodological pilot grants in partnership with our CTSA Pilots Program. These grants promote impactful BERD-related research, and awardees have gone on to publish findings and secure NIH R01 funding, demonstrating the impact of this collaboration. The program conducts a variety of education, training, and mentoring activities to help support students and trainees in developing strong methodological skills. Individual BERD consultations are provided to assist investigators with protocol development, sample size planning, statistical analysis plans, pilot grant reviews, funding, and study design. These consultations often lead to longer-term collaborations with biostatisticians.

Effective evaluation is important in complex translational initiatives like CTSA hubs, as it enables demonstration of impactful implementation, efficient management, and real-word impact [5]. National level studies have evaluated BERD programs across CTSA’s, revealing wide variability in program structure, staffing, and documentation practices, highlighting the need for context-specific evaluation strategies [2]. Research highlights the value stakeholder-centered approaches, integrating qualitative insights and quantitative metrics, as best practice for strengthening evaluation infrastructure and fostering quality improvement across CTSA hubs [6,7]. Prior work has explored the collaborative role of biostatisticians in academic medical centers and emphasized the importance of structured consultation relationships between investigators and statistical experts [8].

The aim of this study was to evaluate the Georgia CTSA BERD consultation program to identify strengths, gaps, and opportunities for improving support services and collaboration across the partner institutions. While prior research has examined BERD program infrastructure and collaborative roles, fewer evaluations have assessed consultation services from both investigator and consultant perspectives within a single CTSA hub. By integrating feedback from both perspectives, this study provides a more comprehensive understanding of how BERD consultations function in practice and identifies opportunities to improve consultation workflows, collaboration, and methodological support across translational research environments.

## Methods

We conducted a multi-perspective evaluation of the Georgia CTSA BERD program from 2024 to 2025. We distributed online surveys to both investigator consultees who received consultations and BERD consultants who provided them.

### BERD consultation surveys

A total of 62 individual investigator-focused BERD consultations took place from January 2024 to January 2025. Consultations are provided free of charge on both scheduled and walk-in bases. Following these consultations, we distributed Qualtrics surveys [9] – collaboratively developed by the Evaluation and BERD teams – via email explaining the evaluation objective. The email offered a $20 Amazon gift card to the first 25 respondents; names were collected for tracking, but data were securely stored and deidentified.

The investigator consultee survey included items on participant demographics, awareness, and use of BERD services, satisfaction and impact, and consultation outcomes. The BERD consultant survey collected data on the type and length of consultations, use of innovative statistical methods, and whether a follow-up consultation was required. Open-ended, free-response questions were included in both surveys to capture context-specific insights, elaborate on quantitative responses, and identify unanticipated themes related to the consultation experience. We used a pragmatic mixed-methods approach, which integrates quantitative and qualitative data to provide complementary perspectives when evaluating complex programs and implementation processes [10]. In this approach, quantitative survey items and open-ended qualitative responses were collected concurrently. Quantitative results were summarized descriptively, and open-text responses were reviewed to identify recurring themes, with integration occurring during interpretation to contextualize and compare findings across data types.

Table 1 describes participant information for both the investigator consultees served in these consultations and the BERD consultants. The “Other Institution” category includes consultees affiliated with collaborating organizations outside the four Georgia CTSA institutions. We received survey responses for approximately half of the investigator consultees and BERD consultants, with some variability across institutions. Investigator consultees from across the Georgia CTSA were matched with BERD consultants based on availability and expertise. Survey responses were submitted by 14 BERD consultants, most of whom completed multiple consultations (1–16 each). The median number of consultations completed per BERD consultant was 3.5 (IQR = 2.25–5.75). Quantitative data were analyzed descriptively using Excel, and open-text responses were reviewed to identify recurring themes and opportunities for improvement.

**Table 1.** BERD consultations and survey respondent characteristics by institution (Jan 2024–Jan 2025)

	Emory	MSM	UGA	Georgia Tech	Other institution	Total
*Investigator consultee*	23	22	5	7	5	62
*BERD consultant*	25	23	7	7	0	62
*Investigator consultee* *survey respondents*	12 (46.0%)	6 (23.0%)	2 (8.0%)	2 (8.0%)	4 (15.0%)	26 (42.0%)
*BERD consultant survey respondents*	10 (29.0%)	18 (53.0%)	5 (15.0%)	1 (3.0%)	0 (0.0%)	34 (55.0%)

*Note*: Percentages represent the proportion of investigator consultee survey respondents (n = 26) or BERD consultant survey respondents (n = 34), depending on the row. BERD = Biostatistics, Epidemiology, and Research Design; Emory = Emory University; MSM = Morehouse School of Medicine; UGA = University of Georgia; Georgia Tech = Georgia Institute of Technology.

## Results

### Individual consultation surveys

Table 2 summarizes survey responses from both investigator consultees and BERD consultants. Investigator consultee respondents spanned trainees to senior faculty, most commonly assistant professors (34.6%) and students (23.1%). Academic degrees also varied, with PhD (36.0%) and MD (32.0%) most frequently, alongside other professional and graduate training (PharmD, DrPH, master’s, bachelor’s). Most investigator consultees accessed BERD support during the early stages of grant applications (48.0%), and services were most often initiated through direct requests to a BERD consultant (38.4%) or via the Georgia CTSA website (42.3%).

**Table 2.** BERD consultation survey- participant responses

Survey question/topic	response	n (%)
Investigator consultee responses		
*How BERD services were accessed*	Georgia CTSA website Contacting BERD staff directly Coordinating center Other	11 (42.3%) 10 (38.4%) 3 (11.5%) 2 (7.7%)
*Stage of research at consultation*	Grant application Unfunded project Post grant award During funded project Other	12 (48.0%) 7 (28.0%) 3 (12.0%) 2 (8.0%) 1 (4.0%)
*Satisfaction with consultation*	Extremely satisfied Somewhat satisfied Somewhat dissatisfied Neither satisfied nor dissatisfied	18 (69.0%) 5 (19.0%) 2 (7.7%) 1 (3.8%)
*How likely to recommend BERD services*	Very likely Likely Somewhat likely Unlikely	17 (65.0%) 4 (15.0%) 4 (15.0%) 1 (4.0%)
BERD consultant responses		
*Length of consultation*	Single session (less than 1 hour) Single session (1-2 hours) Multiple sessions (over few weeks) Multiple sessions (over few months) Ongoing support (over several months)	5 (15.0%) 6 (17.5%) 11 (32.5%) 7 (21.0%) 4 (12.0%)
*Types of assistance*	Data analysis Study design/sample size Study design + data analysis Other	14 (42.0%) 8 (24.0%) 5 (15.0%) 6 (18.0%)
*Was first consult sufficient?*	Yes Further help needed	12 (35.0%) 22 (65.0%)

*Note:* Percentages may not sum to 100 due to rounding, multiple selections, and respondents may not have answered all questions. Georgia CTSA = Georgia Clinical and Translational Science Alliance; BERD = Biostatistics, Epidemiology, and Research Design.

Investigator consultee responses indicated that they were extremely satisfied (69.0%) or somewhat satisfied (19.0%) with the quality and effectiveness of BERD consultations. Although some groups were too small to support meaningful analysis, scores were uniformly high across all subgroups. Most respondents indicated that their statistical needs were met and that the BERD consultants provided support with new or advanced statistical methods. Investigator consultees described consultations as collaborative, timely, and impactful, especially in areas involving advanced statistical methods such as Cox proportional hazards modeling, McNemar’s test, and longitudinal data strategies. One respondent noted, *“She was able to perform Cox-PH modeling and KM curves that would have taken me much longer to perform, with rapid ability to make modifications and improve figures.”*

Open-ended feedback praised the clarity of the support received, and also offered constructive suggestions for improvement. A few Investigator consultees noted gaps in consultant expertise for specific project types, especially for qualitative or mixed-methods research. Others pointed toward limitations when their consultant was unfamiliar with the scientific context of a study. A consultee commented “*She did not understand our project…she could not answer [our questions] because she did not understand the goal*.” Despite challenges, consultees expressed interest in continuing to work with BERD again and supported the expansion of its services. Another consultee added, *“I am happy with the previous consultation and would like to get consultation service in the future*.”

BERD consultants represented all four Georgia CTSA institutions and reported varying levels of consultation activity. The majority of consultations were concentrated at MSM and Emory, with fewer reported at UGA and Georgia Tech. Responses underscored BERD’s value while pointing to needs in efficiency, documentation, and pre-consultation workflows.

BERD consultants described consultations as collaborative and iterative, with many projects requiring more than one session to meet investigator consultees requests (65.0%). Follow-up support was commonly required for complex or evolving projects, including ongoing data collection, grant proposal development, or manuscript preparation. BERD consultants described providing continued support over multiple months, with many citing their involvement in capstone mentorship, funded proposals, and abstract submissions. As one BERD consultant noted, the “*Support extended through 2024 and led to methods development and a capstone project*.” Another consultant noted, “*Multiple follow-ups… they are submitting an abstract related to this work*.”

Consultations mainly addressed standard statistical principles, with a smaller proportion involving advanced methods such as geospatial analysis, propensity score weighting, biomarker sample size planning, and long-term longitudinal modeling. BERD consultants who applied these methods emphasized their importance in enhancing study design and contributing toward methodological development for supporting publication efforts.

Challenges identified by BERD consultants included investigator consultees arriving unprepared, lacking clear project goals, or not providing sufficient data for a productive consultation. Communication delays and missed appointments affected the continuity of support as one consultant shared, “*The requestor did not bring enough information to scope their project*,” another noted “*I did not hear back…my assumption is that she went forward with what she was doing*.”

## Discussion

This evaluation offers insights into the Georgia CTSA BERD program’s role in supporting CTS across a multi-institutional CTSA hub. Feedback from both investigator consultees and BERD consultants highlights high satisfaction and tangible impacts. Investigator consultees described consultations as tailored, timely, and instrumental for study design and grant applications, while BERD consultants emphasized their collaborative nature and sustained value in advancing research quality, publications, and funding. Taken together, examining investigator and consultant perspectives provided a more complete understanding of BERD consultation services than either perspective alone. Investigator consultee responses reflected perceived value, satisfaction, and usefulness of consultations for advancing study design and funding goals. In contrast, BERD consultants highlighted operational realities, including variability in project readiness, challenges in defining scope, and inefficiencies that affected consultation workflows. Considering these perspectives together revealed an important insight that would not be apparent from a single viewpoint: while consultations are experienced as effective and valuable by investigators, the processes to deliver this support can place uneven demands on BERD consultants. This divergence helps explain why high user satisfaction does not equate to optimal efficiency or sustainability of biostatistical services. For CTSA hubs and beyond, this underscores the value of a multi-perspective evaluation approach for identifying improvement opportunities that balance investigators’ needs with sustainable biostatistical capacity.

BERD pilot and conference grants foster methodological innovation, early-career development, and collaborative research by funding early-stage projects and promoting translational impact. The use of services by early-career investigators and trainees confirm that services are reaching BERD’s priority user groups and aligns with past research showing that targeted support enhances productivity and capacity among emerging researchers [5]. Evaluations of CTSA funded pilot grant programs demonstrate that structured support for early career faculty leads to grant success and increased research capacity [11]. Research indicates that biostatistical consultation, grant writing workshops, and mentorship increase the likelihood of junior investigator securing external award funding and producing publications [12,13]. Mentoring and methodological training have been identified as critical for early career investigators navigating their first NIH applications, with CTSA hubs focusing on integrated workforce development programs that combine statistical expertise, mentoring, and team-based research [14,15]. These findings align with prior research highlighting the importance of formal training approaches for collaborative biostatisticians, which emphasize effective communication, interdisciplinary teamwork, and sustained engagement with investigators throughout the research lifecycle [16]. Biostatistics is rapidly evolving, and centralized units benefit researchers by supporting faculty training and fostering regular networking with key biostatisticians [17].

A strength of this evaluation is its integration of quantitative metrics with qualitative stakeholder feedback consistent with best-practice recommendations for biostatistics consultation that emphasize stakeholder engagement, continuous quality improvement, and program adaptation [6,18,19]. These findings are consistent with established best practices for biostatistics consultation, which emphasizes early engagement in study design iterative collaboration, and sustained methodological support throughout the research life cycle. Effective biostatistical consultation often extends beyond single encounters to include follow-up, contextual understanding of the scientific question and clear communication between investigators and biostatisticians [17]. The frequent need for follow-up consultations underscores the collaborative and iterative nature of BERD support, reflecting consultation models that emphasize sustained methodological engagement rather than one-time interactions. Identified challenges, including unclear project goals, and consult-investigator misalignment, mirror issues described in biostatistics consultation research and highlight opportunities for targeted quality improvement [7]. These strategies comport with our findings and could inform efforts to improve consultant availability and consistency across partner institutions [17].

### Limitations

This evaluation included a relatively small sample size, which limits the generalizability of the findings. Investigator consultee responses were voluntary and may reflect a more engaged or satisfied subgroup. Consultant responses were concentrated at one or two institutions, limiting the representativeness across the Georgia CTSA. We did not assess cross-institution collaboration within individual consultations, limiting our ability to evaluate how investigators accessed complementary expertise across partner institutions. Additionally, the evaluation focused on short-term feedback and did not assess long-term outcomes such as publications or grant success. Future evaluations could include larger samples, longer-term outcomes, and qualitative methods like interviews to provide a deeper insight into customer experiences.

### Recommendations and future directions

The Georgia CTSA BERD program plays an important role in advancing CTS by supporting early-career and more experienced investigators across multiple disciplines. Its structure, with BERD faculty spanning Emory, MSM, Georgia Tech and UGA, enables broad access to high quality biostatistical expertise and fosters a collaborative space for addressing complex research questions. Through a range of services including consultations, studio sessions, pilot grant support, educational programming, and an annual forum, the program directly contributes to study designs, more competitive grant proposals, successful research implementation, and improved collaboration.

The program’s flexibility in engaging investigators at any stage of the research process, positions BERD to serve as a scalable support model, enabling it to adapt to evolving methodological needs as projects progress. BERD programs emphasize collaboration and represent a valuable resource for researchers with varying methodological needs. By serving early-career investigators, trainees, and experienced investigators, BERD strengthens the research capacity across institutions, helping to sustain a well-trained translational workforce.

Looking ahead, the program is well-positioned to expand its impact across CTS and beyond. Enhancing long-term engagement strategies such as post-consultation check-ins could improve continuity of support and help downstream outcomes like publication and funding success. Continued investment in emerging areas would help further increase BERD’s relevance and responsiveness to evolving investigator needs. Beyond local program assessment, this evaluation contributes to the CTSA literature by demonstrating a multi-perspective approach to BERD evaluation that integrates both investigator and consultant experiences, offering transferable, practice-oriented insights into how biostatistical support is delivered and optimized within multi-institutional academic research environments. By emphasizing transferable consultation processes rather than program-specific structures, these insights are relevant to BERD programs across CTSA hubs and similar academic research environments. More broadly, these findings reaffirm the central role of biostatistics within the university research infrastructure, where dedicated statistical units are essential for advancing rigorous, innovative, and collaborative science.
